# Development and validation of self-reported line drawings of the modified Beighton score for the assessment of generalised joint hypermobility

**DOI:** 10.1186/s12874-017-0464-8

**Published:** 2018-01-17

**Authors:** Dale J. Cooper, Brigitte E. Scammell, Mark E. Batt, Debbie Palmer

**Affiliations:** 1 Division of Rheumatology, Orthopaedics and Dermatology, School of Medicine, University of Nottingham, Nottingham, England; 2 Arthritis Research UK Centre for Sport, Exercise and Osteoarthritis, University of Nottingham, Nottingham, England; 3 Nottingham University Hospitals NHS Trust, Nottingham, England; 4 School of Applied Sciences, Edinburgh Napier University, Edinburgh, Scotland

**Keywords:** Generalised joint hypermobility, Validation, Self-report

## Abstract

**Background:**

The impracticalities and comparative expense of carrying out a clinical assessment is an obstacle in many large epidemiological studies. The purpose of this study was to develop and validate a series of electronic self-reported line drawing instruments based on the modified Beighton scoring system for the assessment of self-reported generalised joint hypermobility.

**Methods:**

Five sets of line drawings were created to depict the 9-point Beighton score criteria. Each instrument consisted of an explanatory question whereby participants were asked to select the line drawing which best represented their joints. Fifty participants completed the self-report online instrument on two occasions, before attending a clinical assessment. A blinded expert clinical observer then assessed participants’ on two occasions, using a standardised goniometry measurement protocol. Validity of the instrument was assessed by participant-observer agreement and reliability by participant repeatability and observer repeatability using unweighted Cohen’s kappa (k). Validity and reliability were assessed for each item in the self-reported instrument separately, and for the sum of the total scores. An aggregate score for generalised joint hypermobility was determined based on a Beighton score of 4 or more out of 9.

**Results:**

Observer-repeatability between the two clinical assessments demonstrated perfect agreement (k 1.00; 95% CI 1.00, 1.00). Self-reported participant-repeatability was lower but it was still excellent (k 0.91; 95% CI 0.74, 1.00). The participant-observer agreement was excellent (k 0.96; 95% CI 0.87, 1.00). Validity was excellent for the self-report instrument, with a good sensitivity of 0.87 (95% CI 0.81, 0.91) and excellent specificity of 0.99 (95% CI 0.98, 1.00).

**Conclusions:**

The self-reported instrument provides a valid and reliable assessment of the presence of generalised joint hypermobility and may have practical use in epidemiological studies.

**Electronic supplementary material:**

The online version of this article (10.1186/s12874-017-0464-8) contains supplementary material, which is available to authorized users.

## Background

A hypermobile joint is one that exceeds the norm for that individual, taking into consideration age, sex and ethnicity [1]. The term generalised joint hypermobility (GJH) is reserved for use when multiple joints are affected and the particular threshold for defining GJH is reached. The Beighton 9-point scoring system – also referred to as the modified or revised Beighton score – is widely accepted as the method used to define GJH [2]. Estimates of the prevalence of GJH vary widely, ranging from 10% to 30% in the general adult population [3], and 2% and 65% in school children [4, 5]. One explanation for the wide range of prevalence estimates is that the cut-off thresholds used to denote GJH often vary between studies. Large populations studies are required to identify suitable cut-off thresholds on the modified Beighton score; thresholds that are sensitive to age, sex and ethnicity.

The lack of a validated assessment tool for self-reported GJH is a barrier to large-scale epidemiological studies. Although the modified Beighton score is relatively quick, safe and simple to use, it requires a trained observer to conduct the assessment. A practical alternative is the use of self-reported line drawings, based on the modified Beighton score [6]. Simple line drawings have been used successfully in self-reported questionnaires for reporting bodily pain [7] and recording physical traits, such as knee mal-alignment and foot rotation [8], Heberden’s and Bouchard’s nodes [9], and hallux valgus [10]. A valid and reliable self-report instrument of the presence of GJH may have practical use in epidemiological studies examining the association with self-reported outcomes of osteoarthritis [11–15], pain [16], and injury among adults during sporting activity [17–22]. The purpose of this study was to develop and validate a set of line-drawing instruments based on the modified Beighton scoring system for the assessment of self-reported GJH.

## Methods

The study formed part of a cross-sectional study into pain and osteoarthritis that was approved by the Nottingham Research Ethics Committee (Reference No: K13022014). Participants were recruited from a mixed population of local community-derived participants using a study advertisement. Implied consent to participate was obtained from all participants completing the electronic self-report instrument and written consent from those who attended for clinical assessment.

### Development of the self-report instrument

A self-report instrument consisting of five line drawings (left and right elbow extension, knee extension, little finger extension, thumb extension, and trunk flexion) was created to depict the 9-point Beighton score criteria. Version one of the instrument depicted one degree of severity (positive test), and version two depicted two degrees of severity (positive and negative test) for each item in the Beighton score. The instrument including both the instructions and the line drawings underwent pilot testing and a review by a Patient Public Involvement (PPI) panel at Nottingham University Hospitals NHS Trust. The first 30 participants who agreed to take part were enrolled into the pilot study, and were invited to complete version one (*n* = 15) or version two (*n* = 15) of the instrument. The PPI panel consisted of six local residents who responded to a study advertisement. PPI members reviewed and completed the instrument and gave feedback during a focus group interview that was digitally recorded. Interviews were listened to and salient points were transcribed verbatim. Two sets of field notes were cross-referenced with the verbal recording and suitable recommendations were fed back into the redesign of the instrument. These amendments were verified by two research assistants following a review of their field notes, and by returning to the PPI members once more for verification. Four of the five line drawings (elbow extension, knee extension, little finger extension, and trunk flexion) were reconfigured to each include three intervals. The remaining item (thumb opposition) consists of two intervals.

**Fig. 1:**
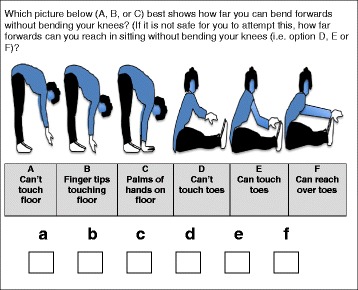
Self-report instrument for assessment of trunk flexion

The first line drawing was created to depict forward flexion of the trunk, with the knees straight, so that the palms of the hands rest flat on the floor. During early pilot testing, it became evident that the use of one and two gradations for trunk flexion yielded a number of false positive test results. PPI members explained that it was unclear if a positive test constituted placing their fingertips on the floor. The line drawing was reconfigured to include three intervals that illustrated: a) the inability to touch the floor (negative test result); b) being able to touch the floor with the fingertips (negative test result); and c) being able to place the palms of the hands flat on the floor (positive test result). A modified version of trunk flexion was also created to enable those who are unable to perform the manoeuver in standing, the opportunity to do so in sitting (see Appendix: Figure 1).

**Fig. 2:**
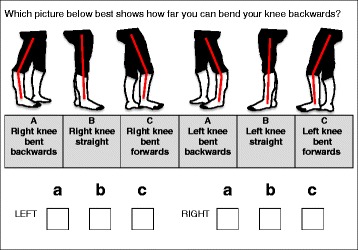
Self-report instrument for assessment of knee extension

The second line drawing depicts extension of the tibiofemoral joint beyond −10 degrees. From a lateral view, a straight aligned knee was drawn with one interval (version one) and two interval changes (version two) of eleven degrees either side to illustrate knee-flexion and knee-extension. During early pilot work, it was noted that participants experienced difficulty in distinguishing between knee-extension range of movement (ROM) of 0–10 degrees (a negative test result), and knee-extension ROM beyond −10 degrees (a positive test result). Thus, three knee intervals were incorporated into the final instrument and two intervals were increased to twenty degrees to help distinguish between them more clearly. The line drawing was reconfigured to illustrate: a) knee-extension ROM of −20 degrees or greater (positive test result); b) knee-extension ROM of 0–19 degrees (negative test result); and c) knee-flexion ROM of 1 degree or greater (negative test result). Following PPI review, a red line was also drawn on the knee line drawing and the subsequent line drawings (thumb, elbow, and little finger) to illustrate the angle at the joints more clearly (see Appendix: Figure 2).

**Fig. 3:**
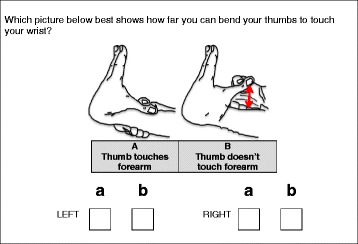
Self-report instrument for assessment of thumb opposition

The third line drawing was created to illustrate the ability to passively extend the thumb and flex the wrist, so that the distal phalanx of the thumb can touch the distal radial side of the adjacent forearm. Following recommendations by PPI members, a line drawing consisting of two intervals depicting: a) the thumb touches the forearm (a positive test); and b) the thumb is unable to touch the forearm (a negative test) was incorporated into the final instrument (see Appendix: Figure 3).

**Fig. 4:**
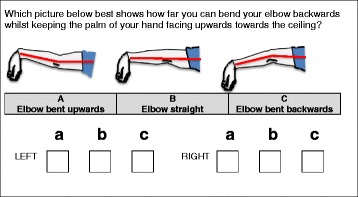
Self-report instrument for assessment of elbow extension

The fourth line drawing illustrates the ability to extend the elbow joint beyond −10 degrees. A single line drawing was created with the elbow in a plane of 0 degrees of extension. Following pilot testing, two further line drawings were created with 11 degrees intervals in either direction. These two intervals were subsequently increased to 15 degrees to help distinguish between them more clearly. Thus, the line drawing consists of three intervals with varying degrees of elbow flexion-extension and is dichotomised into three outcomes: a) elbow flexion (a negative test), b) the elbow in a neutral plane (a negative test), and c) elbow extension (a positive test) (see Appendix: Figure 4).

**Fig. 5:**
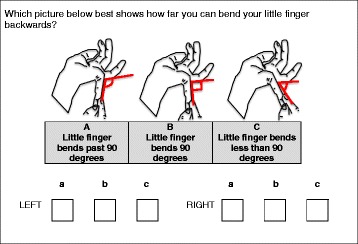
Self-report instrument for assessment of little finger extension

The fifth line drawing was designed to replicate passive extension of the little finger beyond 90 degrees. Pilot testing revealed that line drawings incorporating one and two gradations yielded a high number of false negative test results. PPI members explained it was unclear if extending the little finger to 90 degrees also constituted a positive test. Thus, the final line drawing consists of three intervals to depict: a) the little finger extending beyond 90 degrees (positive test result), b) the little finger extending equal to 90 degrees (negative test result), and c) the little finger extending less than 90 degrees (negative test result) (see Appendix: Figure 5). Each item in the self-report instrument was accompanied by a set of instructions communicating to the participant how the line drawings should be used to determine GJH.

### Validity and reliability of the self-report instrument

The final instrument was validated in 50 participants who twice completed the self-reported instrument online, a fortnight apart. Participants then subsequently attended for a first clinical assessment, and then again one week later for a second clinical assessment. The results of the previous assessment were not made available to the participants. The purpose of the clinical assessment was to determine the level of participant-observer agreement. One examiner completed the self-report instrument using a scoring card and goniometry measurements of the elbow, knee, and little finger were taken using standard practice guidelines [23]. Trunk flexion was assessed using a modified fingertip-to-floor distance (FFD) measurement. Goniometric measurements of the elbow and knee have been shown to have excellent reliability [24, 25]. The FFD has been shown to have excellent inter-test reliability of lumbar spine flexion [26], although it was adjusted to include the participants attempting to place their palms of the hands flat on the floor. So as to ensure that the observer was blinded to the results of the participants, the online self-report data was downloaded only after the clinical assessments had taken place. A second observer, who was blinded to the participants’ results, scored the self-report instrument using data from the observer’s scorecard. Data from each item in the self-report form was reduced by the second independent assessor to either a ‘positive’ or ‘negative’ result for joint hypermobility (JH) based on the Beighton criteria [6]. GJH was measured using a threshold cut-point of 4 of 9 to categorise participants as hypermobile, in line with previous studies on GJH. The results of the previous mechanical goniometry assessment were not made available to the assessor, or to the participants. There was no self-reported change in health status between the distributions of the two self-report forms or clinical assessments.

### Statistical analysis

Validity of the self-report instrument was assessed by calculating the sensitivity, specificity, and the participant-observer agreement. Standard two-by-two tables were formulated to calculate sensitivity and specificity and their 95% confidence intervals (CIs). The Cohen’s unweighted kappa statistic (k) and its 95% CIs were used to calculate repeated measures between the participants and the observer – the reference standard. Reliability of the instrument was assessed by Cohen’s unweighted kappa statistics (k) (95% CI) for participant-repeatability (*n* = 50), and observer-repeatability (*n* = 50), and participant-observer agreement (*n* = 50) at two weekly intervals. Cohen’s kappa statistics were interpreted as follows: < 0 = poor, 0.01–0.20 = slight, 0.21–0.40 = fair**,** 0.41–0.60 = moderate, 0.61–0.80 = substantial, and 0.81–1 = almost perfect [27].

Validity and reliability were assessed for each item in the self-reported JH instrument separately, and for the sum of the total scores. To calculate these outcomes, the JH grade for each item of the instrument was dichotomised as positive (hypermobile) or negative (non-hypermobile) by classifying the most severe grade as present, specifically, category C for elbow extension, category A for extension of the little finger, knee and thumb, and category C and F for trunk flexion in standing or sitting, respectively (see Appendix: Figures 1-5). Analyses were performed using SPSS software version 22.0 and the 95% confidence intervals for Cohen’s kappa were calculated manually.

## Results

### Participant demographics

Fifty participants provided data for the self-reported GJH score reliability and validity assessment. Participants ranged from 20 to 66 years, with a median age of 49. Twenty-two of the participants were male. The prevalence of GJH using the reference standard (i.e. clinical assessment) was 14% defined by a cut-off threshold of ≥ 4/9 on the modified Beighton scale [6]. A total of 78.6% of females were non-hypermobile and 21.4% were hypermobile. This compared with 95.5% of males who were non-hypermobile and 4.5% who were classed as hypermobile. During this validation, the full series of each line drawing depicting hypermobility and non-hypermobility was assessed.

### Validity of self-reported instrument

The values of sensitivity and specificity for the final GJH instrument (version three), along with the results from early pilot testing (version one and two) can be seen in Table 1. All three versions of the instrument appeared to be highly sensitive and specific for right and left thumb extension (Table 1). For trunk flexion in standing, calculated values for sensitivity and specificity were 1.00 (95% CI 0.20, 1.00) and 0.54 (95% CI 0.26, 0.80) for version one, and 1.00 (95% CI 0.31, 1.00) and 0.83 (95% CI 0.51, 0.97) for version two. The use of three intervals (version 3) was highly sensitive (1.00; 95% CI 0.73, 1.00) and specific for trunk flexion (1.00; 95% CI 0.95, 1.00) (Table 1).

**Table 1:** Validity data for the self-reporting line drawing instrument

Self-Report Instrument	Version One		Version Two		Version Three	
	(One-Interval, *n* = 15)		(Two-Intervals, *n* = 15)		(Two-Three Intervals, *n* = 50)	
	Sensitivity	Specificity	Sensitivity	Specificity	Sensitivity	Specificity
	(95% CI)	(95% CI)	(95% CI)	(95% CI)	(95% CI)	(95% CI)
Item-1: Lumbar Sp.	1.00 (0.20, 1.00)	0.54 (0.26, 0.80)	1.00 (0.31, 1.00)	0.83 (0.51, 0.97)	1.00 (0.73, 1.00)	1.00 (0.95 1.00)
Item-1: Lumbar Sp. (sitting)	–	–	–	–	1.00 (0.73, 1.00)	1.00 (0.95, 1.00)
Item-2: Knee R	0.50 (0.09, 0.91)	0.91 (0.57, 0.99)	0.50 (0.09, 0.91)	1.00 (0.68, 1.00)	1.00 (0.82, 1.00)	0.97 (0.90, 0.99)
Item-3: Knee L	0.50 (0.09, 0.91)	0.73 (0.39, 0.93)	0.50 (0.03, 0.97)	0.92 (0.62, 0.99)	0.91 (0.57, 0.99)	0.99 (0.93, 0.99)
Item-4: Thumb R	1.00 (0.40, 1.00)	1.00 (0.68, 1.00)	1.00 (0.31, 1.00)	1.00 (0.70, 1.00)	1.00 (0.80, 1.00)	1.00 (0.94, 1.00)
Item-5: Thumb L	1.00 (0.40, 1.00)	1.00 (0.68, 1.00)	1.00 (0.05, 1.00)	1.00 (0.73, 1.00)	1.00 (0.78, 1.00)	1.00 (0.94, 1.00)
Item-6: Elbow R	0.50 (0.17, 0.83)	0.50 (0.29, 0.71)	0.33 (0.02, 0.87)	0.92 (0.60, 0.99)	0.68 (0.48, 0.83)	1.00 (0.94, 1.00)
Item-7: Elbow L	0.33 (0.02, 0.87)	0.83 (0.51, 0.97)	0.50 (0.09, 0.91)	0.91 (0.57, 0.99)	0.90 (0.72, 0.97)	0.99 (0.91, 0.99)
Item-8: Little Finger R	0.50 (0.03, 0.97)	0.69 (0.39, 0.90)	0.33 (0.02, 0.87)	0.75 (0.43, 0.93)	0.67 (0.39, 0.87)	1.00 (0.95, 1.00)
Item-9: Little Finger L	0.50 (0.03, 0.97)	0.77 (0.46, 0.94)	0.50 (0.09, 0.91)	0.82 (0.48, 0.97)	0.60 (0.27, 0.86)	1.00 (0.95, 1.00)
Overall	0.63 (0.44, 0.79)	0.82 (0.73, 0.88)	0.62 (0.41, 0.79)	0.90 (0.82, 0.95)	0.87 (0.81, 0.91)	0.99 (0.98, 1.00)

*CI* confidence interval

The values for sensitivity for self-report knee extension were high for the right knee (1.00; 95% CI 0.82, 1.00) and left knee (0.91; 95% CI 0.57, 0.99) for the final instrument (version three). The sensitivity for self-report knee extension provided a moderate assessment for both knees (0.50; 95% CI, 0.09, 0.91) for version one of the instrument. These findings were similar for the right knee (0.50; 95% CI 0.09, 0.91) and the left knee (0.50; 95% CI 0.03, 0.97) for version two of the instrument (Table 1; Additional file 1). The sensitivity and specificity for elbow extension and little finger extension both provided a fair-to-moderate assessment during pilot testing (Table 1). The use of three intervals provided for a highly sensitive and specific instrument for extension of both elbows and little fingers (Table 1).

During pilot testing, the sum of each item in the self-reported GJH instrument appeared to provide a valid assessment of GJH. The sum of each item in the final instrument (version three) appeared to be highly sensitive (0.87; 95% CI 0.81, 0.91), and specific (0.99; 95% CI 0.98, 1.00).

### Reliability of the self-reported instrument

The reliability scores of the final GJH instrument (version three) are shown in Table 2. The Cohen’s kappa score for participant-repeatability, observer-repeatability, and participant-observer agreement was perfect for trunk flexion and bilateral thumb extension (see Table 2). Participant-repeatability and observer-repeatability were similar and excellent for extension of the left and right elbow, albeit with a slightly wider confidence interval for the right elbow. The participant-observer agreement was also excellent for the left elbow. Despite the lower score, there was still substantial agreement for the right elbow (Table 2). The observer repeatability was perfect for the right knee and identical to the participant repeatability for the left knee. The participant-observer agreement was excellent for the right knee and slightly lower but still excellent for the left knee (Table 2; Additional file 2).

**Table 2:** Reproducibility and agreement between the self-reporting line drawings and the clinical assessment

	Reproducibility		Agreement
Self-Report Instrument	Participant Intra	Observer Intra	Participant Observer Inter
	k (95% CI)	k (95% CI)	k (95% CI)
Item-1: Lumbar Sp.	1.00	1.00	1.00
Item-1: Lumbar Sp. (sitting)	1.00	1.00	1.00
Item-2: Knee R	0.88 (0.73, 1.00)	1.00	0.95 (0.87, 1.00)
Item-3: Knee L	0.88 (0.86, 1.00)	0.88 (0.86, 1.00)	0.90 (0.76, 1.00)
Item-4: Thumb R	1.00	1.00	1.00
Item-5: Thumb L	1.00	1.00	1.00
Item-6: Elbow R	0.94 (0.81, 1.00)	0.95 (0.84, 1.00)	0.75 (0.60, 0.90)
Item-7: Elbow L	0.95 (0.84, 1.00)	0.95 (0.85, 1.00)	0.90 (0.81, 1.00)
Item-8: Little Finger R	0.88 (0.64, 1.00)	1.00	0.77 (0.59, 0.96)
Item-9: Little Finger L	0.79 (0.39, 1.00)	0.88 (0.64, 1.00)	0.73 (0.48, 0.98)
Beighton Score ≥ 4/9	0.91 (0.74, 1.00)	1.00	0.96 (0.87, 1.00)

< 0 = poor agreement, 0.01–0.20 = slight agreement, 0.21–0.40 = fair agreement, 0.41–0.60 = moderate agreement, 0.61–0.80 = substantial agreement, 0.81–1.00 = almost perfect agreement [27]

Participant-repeatability was excellent for the right little finger, and although it was lower for the left little finger, there was still substantial agreement and considerable overlap of the 95% CIs. The observer-repeatability was perfect for the right little finger and excellent for the left little finger. Observer-participant repeatability was greater for the right little finger with substantial agreement for the left little finger. Numerically the scores for left and right little finger were only 0.04 apart (Table 2). The Cohen’s kappa score for the aggregate scores for each of the items in the self-reported instrument demonstrated excellent participant-observer agreement. Observer-repeatability demonstrated perfect agreement. Compared with the observer-repeatability, participant-repeatability was lower but there was still excellent agreement (Table 2).

## Discussion

The aim of the present study was to develop and validate a set of line-drawing instruments based on the modified Beighton scoring system for the assessment of self-reported GJH. The self-report instrument has high validity and reliability for each item, and also for the sum of the total scores. Importantly the self-report instrument provided strong agreement with expert clinical assessment – the reference standard used in clinical practice. The modified trunk flexion test in long sitting was also comparable to self-reported trunk flexion in standing, and expert clinical assessment in both standing and in long sitting. PPI members reported that the greater number of depictions and the instructions enabled participants to distinguish more clearly between a positive and a negative test result. In this study population, the instrument appears to be sensitive, specific, and reliable and, as such, appears to be suitable for the assessment of GJH for use in patient reported outcome measures.

Previous studies of GJH tend to rely upon undertaking a physical examination [16, 17], which is time consuming, costly and impractical for epidemiological studies of significant size. Alternative methods include the five-part self-report hypermobility questionnaire by Hakim and Grahame [28], which has been validated in a clinical setting but not yet validated in community-based populations. Their questionnaire was more efficient in large epidemiological studies compared with the undertaking of a physical examination, but it wasn’t able to identify isolated hypermobile joints. The ability to compare the results between studies is also hampered with the use of the five-part questionnaire due to the reliance in previous studies on using the Beighton score – the internationally recognised method of determining GJH. Further advantages of using line drawings over the five-part questionnaire are the ability to detect hypermobile joints that are asymptomatic. A self-report instrument has been validated in the assessment of hypermobility in patients with femoroacetabular impingement [29]. According to this instrument, a positive test result for JH is indicated by extension of the little finger to 90 degrees. Yet the true Beighton criterion requires extension of the 5th metacarpophalangeal joint beyond 90 degrees. This instrument describes only one degree of severity to depict each item in the Beighton score. In the present study population, the use of two and three intervals (version 3) was found to have high validity and reliability for correctly identifying GJH when using the self-report instrument.

The current study has some limitations. Firstly, only a single trained observer was used to act as the reference standard, and despite high observer repeatability, no measurement of inter-observer agreement was sought. Secondly, the reference standard for determining validity and reliability was the observer’s clinical assessment using manual goniometry. The use of radiographic measurement improves measurement accuracy, but it is impracticable for many epidemiological studies. Despite this limitation, goniometric measurements have been shown to have excellent intra-rater reliability and inter-rater reliability [24, 25]. A third caveat concerns the use of the Beighton score, including its lack of representation of lower limb mobility. It provides no indication of the severity of joint hypermobility, and associated traits such as flat feet and mild scoliosis. Furthermore, not only do varied cut-off thresholds limit the ability to make cross study comparisons; pauciarticular hypermobility at joints other than those in the Beighton scale go unnoticed [1].

## Conclusions

In summary, the present study findings show that the self-reporting instrument for assessing GJH has validity and reliability. The instrument is comparable to expert clinical assessment using manual goniometry, and would be particularly suited to large epidemiological studies using electronic questionnaires, given the low cost and reduced burden of administration.

### Additional files

Additional file 1: Validity data for the self-reporting line drawing instrument. Validity data for self-reporting instrument. (XLSX 49 kb)

Additional file 2: Reproducibility and agreement between the self-reporting line drawings and the clinical assessment. Reliability data for self-reporting instrument. (XLSX 43 kb)

## Appendix

You will be presented with a series of pictures that relate to how flexible your joints are. We would like you to look at these pictures, and if it is safe for you to do so, we would like you to try and perform the same movement in front of a mirror. Your flexibility may differ between your right and left side so please score each limb separately where applicable.

## Abbreviations

CI: Confidence interval
FFD: Fingertip-to-floor distance measurement
GJH: Generalised joint hypermobility
JH: Joint hypermobility
PPI: Patient Public Involvement
ROM: Range of Movement
